# Patients at the centre after a health care incident: A scoping review of hospital strategies targeting communication and nonmaterial restoration

**DOI:** 10.1111/hex.13376

**Published:** 2021-12-20

**Authors:** Rachel I. Dijkstra, Ruud T. J. Roodbeen, Renée J. R. Bouwman, Antony Pemberton, Roland Friele

**Affiliations:** ^1^ Department of Criminal Law, Tilburg Law School Tilburg University Tilburg The Netherlands; ^2^ Netherlands Institute for the Study of Crime and Law Enforcement Amsterdam The Netherlands; ^3^ Netherlands Institute for Health Services Research (NIVEL) Utrecht The Netherlands; ^4^ Tranzo Scientific Center for Care and Wellbeing Tilburg University Tilburg The Netherlands; ^5^ Breuer & Intraval Research and Consultancy Research Department Groningen The Netherlands; ^6^ Het Wetenschapsbureau Amsterdam The Netherlands; ^7^ Leuven Institute of Criminology KU Leuven Leuven Belgium

**Keywords:** communication, health care incident, hospital strategies, patients, restoration

## Abstract

**Objective:**

This study aimed to provide an overview of the strategies adopted by hospitals that target effective communication and nonmaterial restoration (i.e., without a financial or material focus) after health care incidents, and to formulate elements in hospital strategies that patients consider essential by analysing how patients have evaluated these strategies.

**Background:**

In the aftermath of a health care incident, hospitals are tasked with responding to the patients' material and nonmaterial needs, mainly restoration and communication. Currently, an overview of these strategies is lacking. In particular, a gap exists concerning how patients evaluate these strategies.

**Search Strategy and Inclusion Criteria:**

To identify studies in this scoping review, and following the methodological framework set out by Arksey and O'Malley, seven subject‐relevant electronic databases were used (PubMed, Medline, Embase, CINAHL, PsycARTICLES, PsycINFO and Psychology & Behavioral Sciences Collection). Reference lists of included studies were also checked for relevant studies. Studies were included if published in English, after 2000 and as peer‐reviewed articles.

**Main Results and Synthesis:**

The search yielded 13,989 hits. The review has a final inclusion of 16 studies. The inclusion led to an analysis of five different hospital strategies: open disclosure processes, communication‐and‐resolution programmes, complaints procedures, patients‐as‐partners in learning from health care incidents and subsequent disclosure, and mediation. The analysis showed three main domains that patients considered essential: interpersonal communication, organisation around disclosure and support, and desired outcomes.

**Patient Contribution:**

This scoping review specifically takes the patient perspective in its methodological design and analysis. Studies were included if they contained an evaluation by patients, and the included studies were analysed on the essential elements for patients.

## 1 INTRODUCTION

In 1999, the Institute of Medicine published its landmark publication ‘To err is human’, which showed a high rate of medical error in health care in the United States of America and the need for improved patient safety.^1^ The analysis from ‘To err is human’, together with several medical tragedies worldwide (such as the Bundaberg Hospital Scandal), functioned to draw attention to the need for safe health care.^2^ Institutions, nations and states launched initiatives to improve openness and disclosure after medical error, such as communication‐and‐resolution programmes (CRPs) and apology laws.^3^, ^4^

Scholars have differed in how they distinguish between types of health care incidents and which terminology they have used. Examples include medical error, patient safety incident and adverse event.^1^, ^5^, ^6^, ^7^ Each of these terms contains within it contextual particularities: something that may seem a medical mistake to a patient may be considered a complication in the eyes of a health care professional. From a patient perspective, a broad range of health care incidents are relevant. We have therefore chosen to use the term ‘health care incident’, defined by the Australian Council for Safety and Quality in Health Care as ‘an event or circumstance during health care which could have, or did, result in unintended or unnecessary harm to a person and/or a complaint, loss or damage’.^8^ We use this term to, indeed, include a variety of incidents and errors that involve medical treatment, medication, communication, management, and service or interpersonal skills of health care professionals.

The strategies used by hospitals did not always provide patients and family members (abbreviated to P/F) with a way to come to terms with what had happened.^9^ Some scholars asserted that the patient perspective was completely lacking.^10^ The term ‘strategies’ is used in this article to include all hospital programmes, processes, policies and interventions. The terms can be used interchangeably. One study demonstrated the need among patients to be communicated with openly about a health care incident (i.e., open disclosure, an open discussion between the patient and the health care professional about the health care incident).^11^ The study showed that, despite momentum for open disclosure in Australia, P/F ‘only rarely experienced incident disclosure communication as appropriate and effective’.^11^ This raises the question of how to meet patients' needs after a health care incident, to prevent unnecessary legal proceedings and subsequent costs and additional psychological, physical or financial harm.^4^, ^12^

Dauer and Bismark^13^ distinguished four patient motives for taking legal action after a health care incident: correction; sanction; communication; and restoration. Different strategies after a health care incident link to a variety of these motives. For example, learning programmes and safety culture have a strong link with a patient's desire for correction and prevention.^14^ Disciplinary proceedings or a calamity procedure in turn link to sanctioning either a health care professional or a care facility.^15^ Open disclosure processes and compensation payments, among others, link to communication and restoration. The present study aims to provide a closer look at this last category of strategies, specifically concentrating on strategies without a financial or material component. We call these ‘nonmaterial strategies’: strategies aimed at restoring the harm that was done, using means such as communication, supporting doctors in open disclosure or mediation.

Earlier research has focused on nonmaterial strategies (e.g., when looking at open disclosure^16^); yet, an overview of these strategies is lacking, especially with regard to how P/F evaluated them. This is important because it would allow health care institutions to reason from the patients' point of view and would allow them a voice.^17^ This in turn may prevent unnecessary legal proceedings and may provide an understanding of fundamental aspects that provide for a good strategy. These fundamental aspects can inform future policy and strategies for all hospitals adhering to the words: ‘listening to patients illuminates the way forward’.^4^ This scoping review provides an overview of strategies adopted by hospitals that target effective communication and nonmaterial restoration and P/F's evaluation of these strategies.

## 2 METHODS

A scoping review fitted the aim of this study because it provides an unprecedented overview of studies that deal with nonmaterial, patient‐directed strategies after a health care incident^18^ using a variety of methods.^19^ No scoping review protocol exists, but the review essentially followed the methodological framework with its five stages set out by Arksey and O'Malley^20^ and used the Prisma Scoping Review Checklist (added as Supporting Information Appendix S1 to this study).^21^

### 2.1 Stage 1: Research question

The research questions that guided the review were as follows: what kind of nonmaterial, patient‐centred hospital strategies with a focus on the impact on patients and family members are published? And how do patients and family members evaluate these strategies?

### 2.2 Stage 2: Relevant studies

The relevant studies were identified by searching electronic databases that were relevant to the research topic and searching the reference lists of the included studies (snowballing). Electronic databases covered PubMed, Medline, Embase, CINAHL, PsycARTICLES, PsycINFO and Psychology and Behavioral Sciences Collection, and were systematically searched on 11 June 2019. To inform the search strategy, three key concepts were used based on the research aim (hospital—health care incident—nonmaterial, patient‐centred strategies), supplemented by keywords specific to the search engines (MeSH, Emtree) and synonyms (Thesaurus). Additional words and phrases were used that targeted a variety of potential strategies to ensure a broad focus in our search. The general search string used is shown in Box 1. The specific search strings for all databases are presented in Supporting Information Appendix S2.

Box 1 **Search string used in electronic databases**

The search strategy used the following search string: (hospital OR hospitals) AND (medical error OR medical errors in hospitals OR malpractice OR diagnostic error OR medication error OR adverse event OR patient harm OR professional misconduct OR bad news OR mistake OR bad news OR difficult news OR sad news OR difficult conversation OR unintentional error OR bad news delivery) AND (assessment tool OR plan of action OR truth disclosure OR breaking bad news OR communication strategies OR doctor–patient relationship OR physician–patient relationship OR professional–patient relationship OR complaint OR complaints OR apology OR apologies OR disclosure OR disclosure of medical errors OR patient support OR communication OR communicate OR explain OR explanation OR restorative justice OR restorative OR experience OR reconciliation OR reconcile OR restore OR restoring OR restoration OR mediation OR education OR training and development of employees OR training).

### 2.3 Stage 3: Study selection

For the study selection, this review used inclusion and exclusion criteria and followed an iterative process. Studies were included for analysis when published in English, focused on humans, focused on hospitals as health care institutions (to allow comparability) and published between 2000 and 11 June 2019, because of increased attention to and implementation of nonmaterial strategies. The three key concepts further informed the inclusion of studies. Each included study had to focus on a strategy (including interventions, programmes, processes or policies) *internal to a hospital*, in the *aftermath* of a health care incident, which had a *nonmaterial and patient‐centred focus* and had been *evaluated by P/F*.

Studies were excluded if no full text was available (despite an attempt to contact authors) or if one of the inclusion criteria was not fulfilled. The scoping review specifically excluded studies that lacked an internal hospital strategy or had not been evaluated by P/F. Hybrid studies that included a material subfocus were included. Quality assessment of the studies was not conducted because this scoping review aimed to provide a descriptive overview of the available research.^20^ In consultation with coauthors, we decided to exclude journal articles that were not peer‐reviewed because these studies did not contain relevant information for this study. Grey literature was not searched because no systematic or scoping review had been performed in the past, so the main aim was to outline current academic publications.

### 2.4 Stage 4: Charting the data

Following Arksey and O'Malley, the scoping review proceeded with charting the main themes and important elements of the included studies; these are presented in Table 1 in the Results section. The table includes information on the author, year of publication, study location, main objective, hospital strategy, design/method, sample size, setting and main outcome. To add to the validity of the study and provide a certain level of consultation, three experts from Australia, the United States of America and Europe were asked to assess inclusion and add missing literature. This did not lead to the inclusion of any additional studies.

**Table 1:** Overview of the included studies and characteristics

Study first author/year	Country	Main objective	Strategy	Design/method	Sample size	Setting	Main outcome
Elwy et al. (2014)	USA	Identifying elements of large‐scale adverse event disclosure processes to improve future disclosure	Open disclosure	Semi‐structured interviews	97 Participants: 27 patients and family members, 38 employees, 28 leaders and 4 congressional staff members	9 Veterans Health Administration facilities	Disclosure recommendations, including increased preparation, tailored communication, reduced complexity and changed disclosure language
Etchegaray et al. (2014)	USA	To analyse and understand how patients and family members can help with the analysis of adverse events	Patient involvement in adverse event analysis	Interviews	5 Patients and 4 family members, 6 clinicians and 13 hospital administrators	6 Hospitals	Recommendations on including patients and family members in event analysis, but in a patient‐centred way
Friele and Sluijs (2006)	Netherlands	To investigate patients' expectations of complaints handling in hospitals before the initial conversation	Complaints procedure	Survey	424 Patients	74 Hospitals	Complaints procedures need to include all stakeholders and be open for change to fit patient expectations
Friele et al. (2008)	Netherlands	To investigate how patients experienced the actual complaints handling process and how this relates to their (unmet) expectations	Complaints procedure	Survey	279 Patients	74 Hospitals	Less than one‐third of the patients considered the complaints process to have led to justice. Patient expectations could be better met if there is more attention for impartiality, apologies and providing information on changes made
Gallagher (2009)	USA	To learn more about effective disclosure strategies	Open disclosure	Case study	1 Patient	1 Hospital	Recommendations for open disclosure, such as making trust an active part of disclosure conversations, and the value of involving patients in error analysis
Hyman and Schechter (2006)	USA	To measure the participants' satisfaction with mediation of medical malpractice lawsuits	Mediation	Structured interviews	24 Plaintiffs and the other participants in each mediation	Referral by a hospital cooperation and 2 other NYC agencies	Plaintiffs tend to experience mediation as fair, satisfying and responsive to their interests
Iedema et al. (2011)	Australia	To establish principles for effective disclosure on the basis of the patients' and family members' experiences of disclosure	Open disclosure	Semi‐structured interviews	39 Patients and 80 family members	Participating health services	Disclosure rarely met the expectations of patients and family members, which health care institutions should improve
Iedema et al. (2008)	Australia	To establish what works in open disclosure for patients and health care staff (evaluation of the Open Disclosure Pilot)	Open disclosure	Interviews	23 Patients and family members, and 131 clinical staff	21 Hospitals (where pilot was implemented)	Overwhelming support for open disclosure from both staff and patients
Iedema et al. (2008)	Australia	To map the perceptions of patients and family members with open disclosure and adverse events	Open disclosure	Semi‐structured interviews	15 Patients and 8 family members	21 Hospitals	Open disclosure processes need staff to be sensitive in communication, offer an apology, provide lasting support and assign patients an active role in learning from the health care incident
Langer et al. (2017)	Germany	To assess the feasibility of a patient–teacher medical error disclosure and prevention model	Open disclosure through patient teaching	Mixed method (pre‐ and postsurvey and workshop)	71 Patients and family members and 53 clinicians	2 Hospitals	Patient–educators are feasible and promising.
Maguire et al. (2016)	USA	To evaluate a disclosure policy for large‐scale adverse events	Open disclosure	Semi‐structured interviews	97 Total: 27 patients and family members, 70 with (VA central office) leaders, hospital employees, congressional staff member	9 Hospitals	Key problems identified: timely communication, a supportive culture, follow‐up and sharing lessons learned
Moore et al. (2017)	USA	To explore whether patients and their family members can provide important elements to increase reconciliation through a CRP because of their own experiences	Communication‐and‐Resolution Programme	Semi‐structured interviews	27 Patients, 3 family members, and 10 staff	3 Hospitals	Overall positive experiences with CRPs (18 out of 30 patients and family members); the study shows the importance of communicating safety efforts, and including the right people in the disclosure among other things
Murtagh et al. (2012)	USA	To analyse how patients respond to different financial compensation offers	Disclosure‐and‐Resolution Programme	Online survey using vignettes	2112 Panellists 18 years of age and older	Vignettes based on hospital programmes	Generous compensation offers do not necessarily decrease chances that patients will seek legal advice and can negatively affect patient views on the candour of apologies and disclosure. The health care incident increases chances of changing doctors
Piper et al. (2014)	Australia	To understand patients' and family members' experiences of open disclosure in rural areas and offer recommendations	Open disclosure	Interviews	13 Patients and family members	Mostly the Emergency Department of a local hospital	Challenges for rural hospitals (e.g., lack of resources, delays, distance between services) have an impact on how disclosure policies are implemented
Sorensen et al. (2009)	Australia	To understand how patients and professionals experience open disclosure and how practice can inform policy (through gaining empirical evidence)	Open disclosure	Interviews	15 Patients, 8 family members, 20 nursing staff, 49 medical staff, 50 clinical/administrative staff and 3 policy coordinators	21 Hospitals and health services	The study can guide policy when focusing on five ‘key elements’: initiate disclosure, an apology, actively reasoning from the patient's perspective (including providing information on changes), communication, cultural awareness
Walton et al. (2019)	Australia	To determine the existence of formal open disclosure processes (implementation of national policy) and the experiences of the patients with these processes	Open disclosure	Survey	7661 Participants	New South Wales hospitals	Patients' experiences may be better if they are informed about their right to full disclosure and open disclosure guidelines should be reviewed and drafted for bedside disclosure

Abbreviation: CRP, communication‐and‐resolution programme.

### 2.5 Stage 5: Analysing and reporting the results

In reporting the results, the analysis focused on basic study characteristics, the content of the specific types of hospital strategies and finally on a thematic analysis of patient essentials in these hospital strategies. In the thematic analysis, priority was assigned to the content of the evaluation by P/F and what they considered essential.

## 3 RESULTS

**Figure 1:**
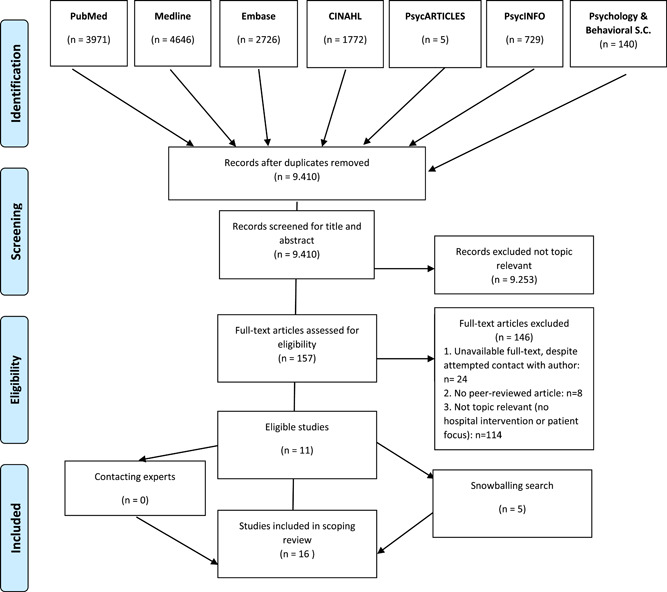
PRISMA 2009 flow diagram—detailed search for this scoping review^43^

Figure 1 presents an overview of the search (flow diagram), yielding a total of 13,989 results—9410 after deduplication. These studies were initially screened on title and abstract by one researcher (R. D.), who made a first selection of 640 studies (e.g., studies about specific drug treatments were excluded). A second researcher (R. R.) screened a random sample of 2% (200 out of 8770 studies) of excluded studies, which did not show conflicts regarding the initial screening.

The initially included 640 studies were screened on title and abstract by two researchers (R. D. and R. R.). Three key concepts (hospital—health care incident—nonmaterial, patient‐centred strategies) were used as criteria for inclusion of articles, resulting in 157 articles. One researcher (R. D.) read the full text of the eligible articles. Two researchers (R. D. and R. R.) then discussed 157 articles based on conflicts (inclusion vs. exclusion), using the knowledge of one researcher (R. D.) who performed the full‐text evaluation. Inclusion criteria were refined to only include studies that comprised (i) an internal hospital programme and (ii) an evaluation by P/F. The discussion led to the inclusion of nine articles. A third researcher (R. B.) reviewed another five studies that were discussed, leading to the inclusion of another two articles and a total of 11 studies. One researcher (R. D.) checked all footnotes of these 11 studies (snowball search), resulting in seven additional eligible studies. Of these seven studies, five were included after consultation with another researcher (R. B.), culminating in a final inclusion of 16 articles.

The Results section contains the study characteristics, the types of hospital interventions and the elements in hospital strategies that patients consider essential.

### 3.1 Study characteristics

The 16 included studies were from the United States of America (seven studies), Australia (six studies), the Netherlands (two studies) and Germany (one study). Ten studies used interviews as their research method, four studies used surveys, one study was a case study and one study used a mixed‐method approach.

All the studies used social‐research design analysis of the hospital strategy by P/F. The sample used to evaluate the hospital strategy consisted of exclusively P/F in eight studies, and of more stakeholders in the other eight studies, such as clinical staff or hospital administrators. The Results section presents the types of hospital interventions and the elements in hospital strategies that patients consider essential.

### 3.2 Types of hospital interventions

Strategies in this scoping review included open disclosure processes (nine studies), CRPs (two studies), complaints procedures (two studies), patients as partners in learning from the health care incident to improve open disclosure and learning (two studies) and mediation (one study).

#### 3.2.1 Open disclosure processes

Nine included studies focused on open disclosure processes in hospitals and have been published between 2008 and 2019.^11^, ^16^, ^22^, ^23^, ^24^, ^25^, ^26^, ^27^, ^28^

##### Hospital implementation of national and regional policies

Four studies targeted the implementation of national or regional policies on open disclosure in hospitals in Australia.^16^, ^24^, ^26^, ^27^ In three studies,^16^, ^24^, ^26^ this implementation was based on the Australian Open Disclosure Standard that aims at ‘more consistent and effective communication after adverse events’.^24^ The fourth study looked into the regional implementation of the NSW Health Open Disclosure Policy.^27^ Two related Australian studies focused on the years after the government endorsement of open disclosure and analysed hospital strategies in terms of patients' experiences.^11^, ^28^

##### Institutional programme

One study analysed one case of a health care incident and institution‐initiated open disclosure in a USA hospital.^23^ The study did not contain information on the specific aims of the hospital strategy, but the author mentioned ‘widespread consensus’ regarding honest and clear disclosure combined with an apology.^23^

##### Open disclosure of large‐scale adverse events

Two studies focused on strategies related to large‐scale adverse event disclosure in the United States of America, evaluating policy by the Veterans Health Administration.^22^, ^25^ Open disclosure by means of this policy was mandatory and aimed at transparency, preserving patients' trust and providing patients with the possibility to do what is needed for their health.^25^

#### Communication‐and‐resolution programmes

Two studies targeted CRPs in the United States of America.^4^, ^29^ CRPs included open disclosure, an explanation, an apology and financial compensation.^29^ The inclusion of a financial component differed from a disclosure programme, although disclosure could also lead to a financial offer by the hospital. Moore's study aimed at exploring P/F experiences with CRPs to see whether they could increase the understanding of how hospital strategies can support reconciliation.^4^ Murtagh's et al.'s^29^ study aimed at exploring how patients responded to different financial compensation offers. The intention of hospitals for implementing CRPs seemed to be a combination of meeting the patient's needs and avoiding lawsuits.^4^

#### 3.2.2 Complaints handling

Two studies focused on complaints handling in Dutch hospitals.^9^, ^30^ These hospitals were obligated by law to have complaints committees, which aimed to ‘warrant easily accessible nonlegal complaints facilities for patients’ and ‘to restore patients’ satisfaction with and trust […] in health care’.^30^ The complaints committees formed a bridge between informal patient support and formal legal procedures and were supposed to provide an independent review of the situation.^9^, ^30^

The first study addressed P/F expectations about the complaints handling procedure before any initial conversations.^30^ The other study compared findings from the first study to new quantitative data on patients' actual experiences with a concluded complaint procedure.^9^

#### 3.2.3 Patients as partners

The review included two studies that assigned P/F an active role in improving open disclosure and preventing health care incidents.^31^, ^32^ The first strategy incorporated P/F as teachers in medical error disclosure and prevention, and aimed to improve health care professionals' communication skills to become more patient‐centred and assign patients an active voice.^31^ The other study addressed the inclusion of P/F in medical error event analysis and disclosure and aimed to investigate and prevent health care incidents, but also to support the healing process of P/F by involving them in the process.^32^

#### 3.2.4 Mediation

The final study discussed mediation.^33^ Mediation was defined as a ‘confidential, voluntary process in which an impartial, third party—the mediator—helps participants negotiate their differences […]’ and it could lead to a binding contract.^33^ The main aim of mediation was ‘to resolve medical malpractice lawsuits’.^33^

### 3.3 Patient essentials in hospital strategies

Despite the variability of strategies, an analysis on the basis of P/F evaluation allowed for extraction of, what we have called, patient essentials from all included studies: elements in hospital programmes that patients considered essential. Each included study was analysed on the basis of what P/F highlighted in their evaluation of the several strategies. The essentials related to three overarching domains: interpersonal communication, organisation of strategies and desired outcomes.

#### 3.3.1 Interpersonal communication

##### Open communication

P/F emphasized the importance of communicating openly and face to face in open disclosure and in the majority of the complaints procedures.^27^, ^28^, ^30^ P/F valued shared dialogue^11^, ^27^ with the health care professional that was involved in their care.^4^, ^24^, ^26^, ^28^ P/F considered continuous^16^, ^23^, ^32^ and consistent communication important.^16^, ^26^ P/F highlighted the importance of hospitals providing information about the (large‐scale) adverse event.^11^, ^22^, ^23^, ^24^ P/F expected the health care professional to be prepared and to explain how this event could occur.^11^, ^30^ One study mentioned the value of tailoring disclosure to the individual if there are pre‐existing conditions, for example, PTSD.^22^

During communication, considerable importance was given to *how* the health care professional conducted the conversation. P/F in complaints procedures indicated that the health care professional should be respectful and candid^30^ and that he/she asked the P/F what they expected from the conversation.^16^, ^26^ P/F emphasized that the health care professional should listen and that there should be room to express emotion.^4^, ^23^, ^24^ P/F described ‘a human approach’^27^ and ‘strong communication skills’ as important.^25^ P/F also appreciated linguistic and cultural sensitivity from health care professionals.^24^, ^26^ P/F indicated that they appreciated suitable language^4^: using the word ‘reconciliation’ rather than ‘resolution’,^4^ using nontechnical language^32^ or adapting language suitable for a phone call as some written words can cause stress.^22^

P/F mentioned pitfalls regarding open communication, for instance, inadequate preparation of staff, and a lack of empathy.^11^, ^27^ P/F sometimes felt that they had to push for open disclosure, for example, by involving the media.^11^, ^16^, ^28^ The importance of social context became clear for P/F from rural areas. Tight social ties could have prevented P/F from asking for open disclosure.^28^ In addition, P/F mentioned a lack of communication between hospitals.^28^ Furthermore, P/F did not appreciate open disclosure being initiated by a letter,^28^ preferring a phone call to a letter,^25^ while P/F involved in a CRP preferred a letter to a phone call.^4^

##### Apology or expression of regret

Interpersonal communication also included an apology. P/F emphasized the value of an apology in the evaluation of open disclosure, CRPs and complaints procedures. P/F considered it important that health care professionals admitted that an error was made^30^ and apologized.^11^, ^23^, ^24^, ^32^ One study on collaborative learning highlighted the value for P/F of a sense of accountability.^31^ Another study stipulated that the benefit of apologies made to P/F was strongly dependent on the identity and perceived candour of the one making the apology.^26^

#### 3.3.2 Organisation of strategies

##### Appreciation for formal open disclosure

Some open disclosure studies suggested that P/F preferred to have a formal^28^ open disclosure process, especially but not limited to situations where a health care incident had severe consequences.^24^ One study mentioned an ‘appropriate level of formality’.^11^ This level of formality allowed P/F to properly prepare for disclosure and to be sure that it took place. P/F in one study considered a formal approach to occur when they were taken seriously and communication was proper, which they considered a sign of respect.^16^

The blur between informal and formal disclosure sometimes confused patients as to whether open disclosure actually came about.^26^ In one study, almost half of P/F considered disclosure insufficiently formal.^16^ In another study, however, most patients indicated that they experienced an informal open disclosure conversation that diminished anger and a feeling of dishonesty.^27^ In addition, Friele et al.^9^, ^30^ showed that too much formality in formal complaints procedures might distract from the genuine conversation with the health care professional.

##### Support

P/F considered support important—to ‘get the right people in the room’.^4^ The importance of the presence and support of specific people was apparent for CRPs and open disclosure processes, but also in programmes that included patients to improve disclosure.^32^ The attending health care professional should play a leading part in any initial disclosure and P/F preferred to have a support person^11^, ^24^, ^26^ with them during meetings regarding medical injury, for example, an attorney.^4^

In addition, P/F identified needs specific to them, such as having a health care professional during open disclosure that was sensitive to the patients' expectations and (cultural and linguistic) context, and who had been involved in their previous care.^24^ P/F did not appreciate being prevented from meeting the staff responsible for their care.^16^, ^23^ One CRP study showed that a positive impact could be made if P/F were contacted on the anniversary of the event to inform them on hospital improvements and to let them know they were not forgotten.^4^

P/F in one study highlighted insufficient follow‐up support related to open disclosure, for example, because it only encompassed one meeting or because P/F did not continuously have the same contact person.^11^ Another study showed that less than 25% of the interviewed patients felt supported during open disclosure, and were seldom asked about their needs.^26^

#### 3.3.3 Desired outcomes

##### Investigation, the need for change and prevention

P/F wanted to be informed about investigative actions^24^ and changes made^16^ to make sure a similar event would not happen again^11^, ^30^ in complaints procedures, open disclosure and CRP programmes. The studies of complaints procedures further indicated that P/F considered the hospital responsible to make changes.^9^, ^30^ P/F expected the complaints committee to investigate the medical injury, to provide validation and recommendations.^30^ P/F involved in the complaints procedure were particularly disheartened by the lack of health care professionals admitting error and lack of changes made.^9^

##### Information (about prevention), closure and rebuilding trust

Preparation for, and explanation during, open disclosure processes facilitated closure for P/F, especially if P/F's concerns had been addressed.^11^ P/F specifically mentioned the significance of disclosure to resolve a health care incident.^16^ However, P/F mentioned that open disclosure processes did not always equal proactive, actual disclosure^11^, ^16^, ^28^ and in one study identified a lack of closure.^11^

P/F in several studies also mentioned the importance of rebuilding trust^22^, ^23^, ^25^ and for the hospital to provide information about future actions.^9^, ^30^, ^32^ Regardless, P/F in a study of open disclosure and of a CRP highlighted insufficient attention paid to providing such information.^4^, ^11^

#### 3.3.4 The need for financial compensation

P/F also indicated financial needs in studies of CRPs, mediation and some of the open disclosure processes. Many USA studies and some Australian studies demonstrated financial motivation on the part of P/F.^4^, ^16^, ^23^, ^26^, ^29^, ^33^ P/F in the other studies made no mention of financial considerations.

The majority of P/F that evaluated a CRP emphasized the desire for financial assistance regarding their immediate needs.^4^ Another CRP study showed that most people want financial compensation, indicating it unlikely that CRPs without a financial component would lead to closure for patients.^29^ P/F in one United States of America^23^ and two Australian studies regarding open disclosure also considered it important to discuss finances^26^ and to receive an offer for tangible support.^16^

However, a high financial compensation offer could discredit the truthfulness of an apology and not meet the patient's wishes—‘money offers change the tenor of patients' view of disclosure and apology’.^29^ This study further showed that despite a CRP aiding resolution, the relationship between patients and health care professionals could deteriorate regardless of the CRP. Finally, the mediation study suggested that plaintiffs and other participants in mediation generally considered this intervention to be ‘fair, satisfying, and responsive to their interests’.^33^ However, some of the plaintiffs (3 out of 12) felt pressured into the mediated agreement.^33^

## 4 DISCUSSION

This scoping review showed that providing a sensitive discussion after a health care incident and a suitable response is not straightforward. It is important to address the individual needs of each P/F and health care professional, particularly regarding interpersonal communication, organisation of strategies and safeguarding outcomes. Below, we reflect on these patient essentials and link them to the hospital strategies and their goals outlined in the Results section.

### 4.1 Interpersonal communication

Substantial attention is paid to the *how* and *with whom* of interpersonal communication. Some of the aspects highlighted by P/F reiterated findings from previous studies, such as openness about the medical error^34^ and the importance of an apology.^12^, ^35^

The importance that patients placed on interpersonal communication corresponded to the goals of open disclosure processes. They aimed for open discussion, transparency, better communication, an apology and preserving trust. In a comparable sense, CRPs aimed to meet the patient's needs, and complaints procedures aspire to provide an independent, nonlegal process to repair the patients' trust. The appreciation of the aforementioned goals is evident in the evaluation by P/F. This is reflected, for example, in the substantial appreciation of sensitive and shared dialogue, ‘a human approach’^27^ and attention paid to the perspective of P/F. These elements seem to result in rebuilding the relationship between P/F and health care professionals and rebuilding trust.

A new finding is the specific and detailed preferences that P/F can have for using specific words.^4^, ^22^, ^32^ For example, ‘reconciliation’ is better than ‘resolution’ and P/F prefer nontechnical language.^4^, ^32^ Also, words like ‘resolved’ are to be avoided, since P/F emphasized that for them, the situation is never resolved.^17^

### 4.2 Organisation of strategies

The second essentiality mentioned by P/F concerns the organisation of hospital strategies and getting ‘the right people in the room’.^4^ P/F considered it of utmost importance that adequate and suitable support is available to them during both an open disclosure process and a CRP. The organisational aspects are distinct from the aims of the hospital strategies because they focused on the strategy's design, not the outcome.

Interestingly, P/F appreciated a more formal approach in open disclosure processes, though what such a formal meeting entailed varies between studies. Formality can mean format (AODS), but P/F consider formality to be the extent to which you are taken seriously.^16^ In any regard, a certain level of formality or preparedness seems to signify respect and provides the opportunity for P/F to prepare for the meeting. However, other studies indicated that patients prefer an informal complaints procedure by a complaints officer over a more formal process with a complaints committee.^36^, ^37^ This idea is addressed in one of the studies on the complaints procedure: Formality should not preclude an open conversation with the involved health care professional.^9^

### 4.3 Outcomes

Finally, P/F have certain desired outcomes: investigation, making changes, prevention, information, closure and financial compensation. Several of the outcomes highlighted by P/F—such as investigation and prevention—link back to underlying goals of particular hospital strategies.^24^, ^26^, ^27^, ^32^ Some of these strategies also aimed to accomplish closure, healing and rebuilding trust^25^, ^30^, ^32^ aside from providing a financial reparation.^25^

Generally, most patients who experienced a health care incident desired quality improvement and change so that a similar event will never happen again. Studies showed that P/F considered the improvement of care to be most important.^38^, ^39^, ^40^ However, studies also showed that only a minority of P/F received feedback on changes made to clinical practice.^39^, ^41^ This scoping review confirms this finding.^4^, ^9^, ^11^, ^30^, ^32^ One intriguing finding regarding the appreciation of financial outcomes by P/F is that the more generous an offer of compensation, the more P/F considered the apology to be serving self‐interest. However, this finding did not lead to an increase in financial claims and malpractice lawsuits, which is consistent with a recent study that showed no increased liability and new claims for operating CRPs.^42^

### 4.4 Methodological considerations

The review has some methodological considerations that need mentioning. To ensure comparability, health care institutions were limited to hospitals. The study only included articles written in English, and articles were not selected based on the methodological quality of the studies. In addition, the study did not explore grey literature. Furthermore, there was considerable overlap between the data used for several of the included studies. Seven of sixteen studies could be traced back to three empirical databases, which reduced potential dispersion. In addition, institutional and cultural differences could influence the type of hospital strategy and how P/F evaluated them, but this has not been explored in the current analysis. Lastly, many of the studies that addressed patients' perceptions of open disclosure were not based on a particular hospital strategy, but rather on a general strategy evaluation. Therefore, these studies could not be included, though they might have provided interesting insights. Consequently, the rigorous approach in this review might have unintentionally excluded interesting studies. In future research, an additional review targeting general strategies as well as grey literature would be recommended.

## 5 CONCLUSION

This scoping review revealed a multitude of nonmaterial, patient‐centred hospital strategies after a health care incident. Future policy and hospital strategies should focus on three main domains to meet patients' needs: (1) interpersonal communication, (2) support and a certain level of formality and (3) fulfilling desired outcomes. P/F in the included studies appreciated openness, good communication, attention to detail and an adequate support system. Also, P/F indicated that the informing of patients about changes made in clinical practice to prevent recurrence was often lacking. Lastly, hospitals dealing with financial compensation offers should be sensitive to the way these offers can reflect on other forms of nonmaterial restoration, such as authenticity of an apology.

## CONFLICT OF INTERESTS

The authors declare that there are no conflict of interests.

## Supporting information

Supplementary information.

Supplementary information.

## Data Availability

The data that support the findings of this study are available on request from the corresponding author. The data are not publicly available due to privacy or ethical restrictions.
