# Supplementary material for: Patients at the centre after a health care incident: A scoping review of hospital strategies targeting communication and nonmaterial restoration
Source: Health Expect. 2021 Dec 20;25(1):264–75. doi: 10.1111/hex.13376 (PMC8849248; doi:10.1111/hex.13376)
Supplement: Supplementary file 2 — Supplementary information. [file HEX-25-264-s001.docx]

**Electronic searches for Scoping Review:**

***Patients at the center after a health care incident: a scoping review of hospital strategies targeting communication and non-material restoration***

**Total: 13689 hits**

**Pubmed search June 11, 2019**

**3971 hits: filters – title/abstract, 2000-2019, human, English**

((((((((((((((((((((((((((("Inservice Training"[Mesh] OR "assessment tool"[Title/Abstract]) OR "plan of action"[Title/Abstract] OR "Truth Disclosure"[Mesh]) OR "Teach-Back Communication"[Mesh]) OR "Professional-Family Relations"[Mesh] OR "Professional-Patient Relations"[Mesh] OR "Physician-Patient Relations"[Mesh] OR "Professional-Patient Relationship"[Title/Abstract] OR complaint[Title/Abstract] OR complaints[Title/Abstract] OR apology[Title/Abstract]) OR apologies[Title/Abstract]) OR disclosure[Title/Abstract]) OR "patient support"[Title/Abstract]) OR "breaking bad news"[Title/Abstract]) OR "truth disclosure"[Title/Abstract]) OR "restorative justice"[Title/Abstract]) OR "doctor patient relationship"[Title/Abstract]) OR "communication strategies"[Title/Abstract]) OR "physician-patient relationship"[Title/Abstract]) OR communication[Title/Abstract]) OR communicate[Title/Abstract]) OR explain[Title/Abstract]) OR explanation[Title/Abstract]) OR restorative[Title/Abstract]) OR experience[Title/Abstract]) OR reconciliation[Title/Abstract]) OR reconcile[Title/Abstract]) OR restore[Title/Abstract]) OR restoring[Title/Abstract]) OR restoration[Title/Abstract]) OR mediation[Title/Abstract]) OR education[Title/Abstract]) OR training[Title/Abstract]) AND ((((((((((((("Medical Errors"[Mesh] OR "Malpractice"[Mesh]) OR "Patient Harm"[Mesh]) OR "Professional Misconduct"[Mesh]) OR "Diagnostic Errors"[Mesh] OR "Medication Errors"[Mesh] OR "medical error"[Title/Abstract] OR "diagnostic error"[Title/Abstract] OR "medication error"[Title/Abstract]) OR "adverse event"[Title/Abstract]) OR malpractice[Title/Abstract]) OR "bad news"[Title/Abstract]) OR "sad news"[Title/Abstract]) OR "difficult conversation"[Title/Abstract]) OR "difficult news"[Title/Abstract]) OR "bad news delivery"[Title/Abstract]) OR "patient harm"[Title/Abstract]) OR mistake[Title/Abstract]) OR "professional misconduct"[Title/Abstract] OR "unintentional error"[Title/Abstract] AND (("Hospitals"[Mesh] OR hospital[Title/Abstract]) OR hospitals[Title/Abstract]) AND (("2000/01/01"[PDAT] : "2019/12/31"[PDAT]) AND "humans"[MeSH Terms] AND English[lang])

**EMBASE search June 11, 2019**

**2726 hits: filters – title/abstract, human, 2000-2019, English**

(hospital:ab,ti OR hospitals:ab,ti OR 'hospital'/exp) AND ('medical error':ab,ti OR 'medical errors in hospitals':ab,ti OR 'adverse event':ab,ti OR malpractice:ab,ti OR 'bad news':ab,ti OR 'sad news':ab,ti OR 'difficult conversation':ab,ti OR 'difficult news':ab,ti OR 'unintentional error':ab,ti OR 'bad news delivery':ab,ti OR 'patient harm':ab,ti OR mistake:ab,ti OR 'professional misconduct':ab,ti OR 'diagnostic error':ab,ti OR 'medication error':ab,ti) AND ('assessment tool':ab,ti OR 'plan of action':ab,ti OR 'truth disclosure':ab,ti OR 'disclosure of medical errors':ab,ti OR complaint:ab,ti OR complaints:ab,ti OR apology:ab,ti OR apologies:ab,ti OR disclosure:ab,ti OR 'patient support':ab,ti OR 'communication strategies':ab,ti OR 'breaking bad news':ab,ti OR communication:ab,ti OR communicate:ab,ti OR explanation:ab,ti OR explain:ab,ti OR 'restorative justice':ab,ti OR restorative:ab,ti OR experience:ab,ti OR reconciliation:ab,ti OR reconcile:ab,ti OR restore:ab,ti OR restoring:ab,ti OR restoration:ab,ti OR mediation:ab,ti OR education:ab,ti OR training:ab,ti OR 'professional-patient relationship':ab,ti OR 'doctor patient relationship':ab,ti OR 'physician-patient relationship':ab,ti OR 'apology'/exp OR 'restorative justice'/exp) AND [english]/lim AND [humans]/lim AND [embase]/lim AND [2000-2019]/py

**EBSCO HOST search June 11, 2019, specific for search engine CINAHL**

**1772 hits: filters – only abstract, human, English, 1-1-2000 to 31-12-2019**

(MW hospital OR AB hospital OR AB hospitals) AND (AB assessment tool OR AB plan of action OR AB ( truth disclosure or breaking bad news ) OR AB communication strategies OR AB doctor patient relationship OR AB physician-patient relationship OR AB professional patient relationship OR AB complaint OR AB complaints OR AB apology OR AB apologies OR AB disclosure OR AB disclosure of medical errors OR AB patient support OR AB communication OR AB communicate OR AB explain OR AB explanation OR AB restorative justice OR AB restorative OR AB experience OR AB reconciliation OR AB reconcile OR AB restore OR AB restoring OR AB restoration OR AB mediation OR AB education OR AB ( training and development of employees ) OR AB training) AND (AB medical error OR AB medical errors in hospitals OR AB malpractice OR AB patient harm OR AB professional misconduct OR AB diagnostic error OR AB medication error OR AB adverse event OR AB bad news OR AB mistake OR AB ( bad news or difficult news or sad news or difficult conversation ) OR AB unintentional error OR AB bad news delivery)

**EBSCO HOST search June 11, 2019 specific for search engine MEDline, PSYCarticles, psycINFO, Social and Behavioural Sciences Collection**

**5520 hits: filters – 2000-2019, human, English**

**Hits specified to different search engines: MEDline [4646], PSYCarticles[5], psycINFO[729], Social and Behavioural Sciences Collection[140]**

(MW hospital OR AB hospital OR AB hospitals) AND (AB assessment tool OR AB plan of action OR AB ( truth disclosure or breaking bad news ) OR AB communication strategies OR AB doctor patient relationship OR AB physician-patient relationship OR AB professional patient relationship OR AB complaint OR AB complaints OR AB apology OR AB apologies OR AB disclosure OR AB disclosure of medical errors OR AB patient support OR AB communication OR AB communicate OR AB explain OR AB explanation OR AB restorative justice OR AB restorative OR AB experience OR AB reconciliation OR AB reconcile OR AB restore OR AB restoring OR AB restoration OR AB mediation OR AB education OR AB ( training and development of employees ) OR AB training) AND (AB medical error OR AB medical errors in hospitals OR AB malpractice OR AB patient harm OR AB professional misconduct OR AB diagnostic error OR AB medication error OR AB adverse event OR AB bad news OR AB mistake OR AB ( bad news or difficult news or sad news or difficult conversation ) OR AB unintentional error OR AB bad news delivery)
